# Thromboangiitis obliterans in two brothers

**DOI:** 10.3892/etm.2013.1160

**Published:** 2013-06-14

**Authors:** QI-LIN LI, DAN-HUA HE, YONG-HUA HUANG, MU NIU

**Affiliations:** Department of Dermatology, Fourth Affiliated Hospital of Jinan University, Guangzhou Red Cross Hospital, Guangzhou, Guangdong 510220, P.R. China

**Keywords:** erythema nodosum, thromboangiitis obliterans, case report

## Abstract

Two brothers (case 1 and case 2) with erythema nodosum were diagnosed with thromboangiitis obliterans (TAO). The patients were treated with compounds including Danshen Dripping Pills, Fufang Danshen Diwan and Salvia tetramethylpyrazine. The patients were also treated with fibro-blast growth factor to promote epidermal growth and Bayaspirin enteric-coated tablets to reduce platelet aggregation. The polysaccharide nucleic acid fraction of Bacillus Calmette-Guérin and compound glycyrrhizin tablets were taken to improve immune function. Following treatment, case 2 had reduced pain levels in the left foot. The ulcer on the first toe of the left foot had decreased in size, with a reduction in pus secretions and inflammation. Case 1 demonstrated a reduction in pus secretion from the ulcer. However, the area of the ulcer had increased, spreading to the fifth toe with gangrene. A tendon had become exposed on the right foot, which was broken and induced severe pain.

## Introduction

Thromboangiitis obliterans (TAO), or Buerger’s disease, is characterized by chronic occlusive segmental lesions of the peripheral blood vessels (1). von Winiwarter provided the first description of a patient with TAO in 1879 (2). In 1908, Buerger produced a detailed description of the pathological findings of the amputated limbs of patients diagnosed with TAO (3). TAO mainly affects small- and medium-sized arteries and veins of the upper and lower extremities. There are a limited number of case reports describing the influence of the cerebral and coronary arteries, the aorta, the intestinal vessels and even multiple-organ involvement in TAO. When TAO is considered to have been identified in an unusual location, the diagnosis may be confirmed only by the observation of acute-phase lesions in the histopathological examination (4,5). TAO occurs worldwide, with a high incidence in the Middle and Far East, and a low incidence in South America and Eastern Europe (6).

It is considered that tobacco, immune system disorders and genes are the three main contributors to TAO (7). The main pathological findings for the diagnosis of TAO are thrombus formation and fibrosis in the blood vessel walls and the perivascular area. TAO develops slowly and occurs periodically, with symptoms including ischemic pain, intermittent claudication and skin ulcers. Symptoms of thrombophlebitis and Raynaud’s phenomenon have been identified in 40% of patients previously diagnosed with TAO (8,9). The treatment of TAO is challenging, due to high disability rates; however, the main treatment methods include microcirculation improvement, and pain and claudication control (10). A limited number of studies concerned with familial TAO exist worldwide, as this form of the disease is rare. This case report describes two brothers who were diagnosed with TAO. The present study was approved by the Guangzhou Red Cross Hospital Medical Ethics Committee, Guangzhou, China. Written informed consetn was obtained from the patients.

## Case reports

### 

#### Case 1

A 33-year-old male was diagnosed with erythema nodosum in both legs. The disease had occurred repeatedly within the last 8 years, and the patient’s symptoms had increased in severity during the past 8 months.

**Figure 1.**
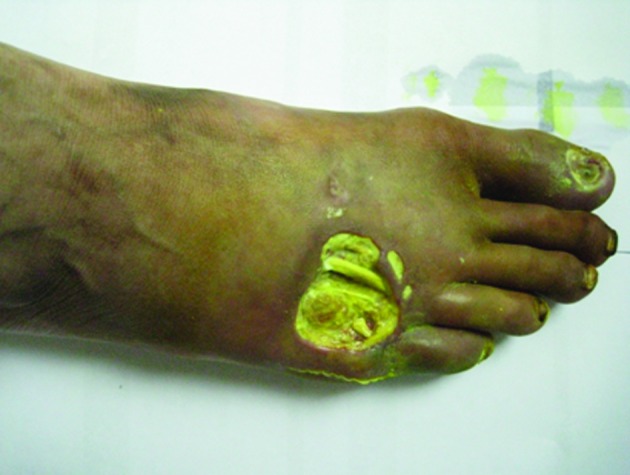
Case 1: The ulcer (4×5 cm) and yellow purulent discharge secretion on the top of the right foot. The fifth toe of the right foot is swollen and infected.

Eight years earlier, the patient had been diagnosed with erythema nodosum in the bilateral lower leg extensors and the dorsum of the right foot, without evident causes. Following a biopsy of the skin tissue from the right foot, the patient was diagnosed with erythema nodosum. The patient’s symptoms were marginally relieved and the tubercles partially disappeared following immune suppression treatment with methotrexate (MTX), triptriolide (TII), cyclophosphamide, prednisolone and methylprednisolone, as well as microcirculation improvement treatment with Xueshuantong tablets and Salvia tetramethylpyrazine. Eight months ago, inflammation had been identified on the first and fifth toes, as well as a secretion of pus, which was diagnosed as paronychia. Following the removal of the first toenail, the severity of the patient’s symptoms increased. An ulcer developed on the right foot, which gradually increased in size. Symptoms such as arthralgia and fever did not occur. The patient had tested positive for tuberculosis in a purified protein derivative (PPD) test five years ago, but tuberculosis was not confirmed. Therefore, the patient was treated with antituberculosis preventive therapy. The patient was diagnosed with hepatitis B, due to a liver function test demonstrating alanine aminotransferase (ALT) levels >1,000 U/l. Subsequent medical intervention with compound glycyrrhizin tablets and diammonium glycyrrhizinate capsules resulted in the normalization of ALT levels. The patient’s medical history did not include hyper-tension, diabetes, coronary heart disease, chronic gastritis, systemic lupus erythematosus (SLE), dermatomyositis, scleroderma, drug allergy, trauma or blood transfusions. The patient had a 10-year history of smoking 5–8 cigarettes per day, and did not abuse alcohol. The physical examination results revealed normal vital signs. The erythema had decreased in size, leaving a small level of pigmentation on the skin; however, the ulcer had increased in size (to 4×5 cm), with partial tendon exposure and a reduction in the yellow purulent discharge. The fifth toe of the right foot was swollen and infected (Fig. 1). In addition, the skin temperature of the right foot was low and the pulse in the top of this foot was weak. Examination identified low levels of touch sensitivity in the right foot.

#### Case 2

**Figure 2.**
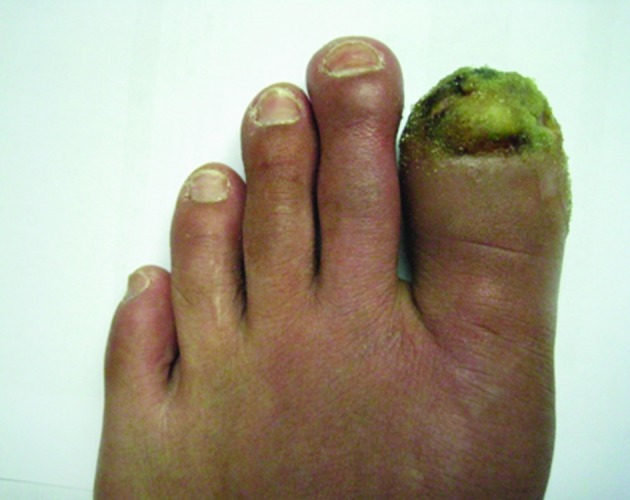
Case 2: The nail of the first toe is broken.

**Figure 3.**
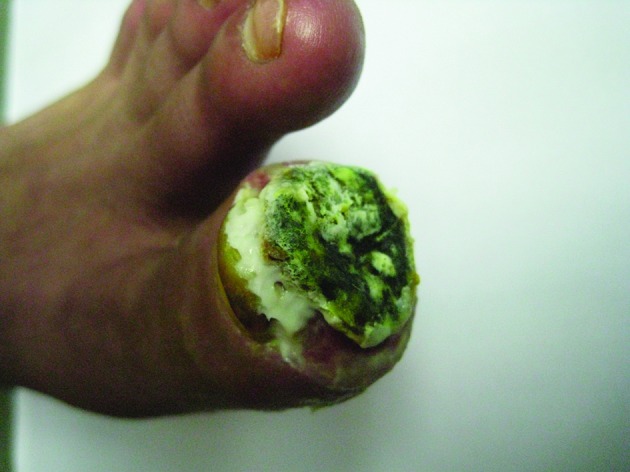
Case 2: The ulcer on the toe, which is partially black with secretion.

A 29-year-old male had been diagnosed with erythema nodosum 3 years earlier, and this condition had become increasingly severe in the past 5 months. Three years ago, the patient was diagnosed with erythema nodosum on the left shank and the top of the left foot. This was accompanied by numbness and pain in the feet, without evident reason. Following hormonal and microcirculation therapy, the symptoms of erythema nodosum decreased. Five months ago, the patient experienced pain in the first toe of the left foot, and partial inflammation and an ulcer were observed. The patient had a 4-year history of smoking 3–5 cigarettes per day. The physical examination revealed normal vital signs. The erythema nodosum on the left shank had disappeared; however, the nail of the first toe on the left foot had become broken and an ulcer was identified on the toe. The ulcer was partially black with a secretion of pus (Figs. 2 and 3). Furthermore, the skin temperature of the foot was low, and the pulse in the top of the foot was weak. An examination identified low levels of touch sensitivity in the left foot.

### Laboratory examination

#### Case 1

**Figure 4.**
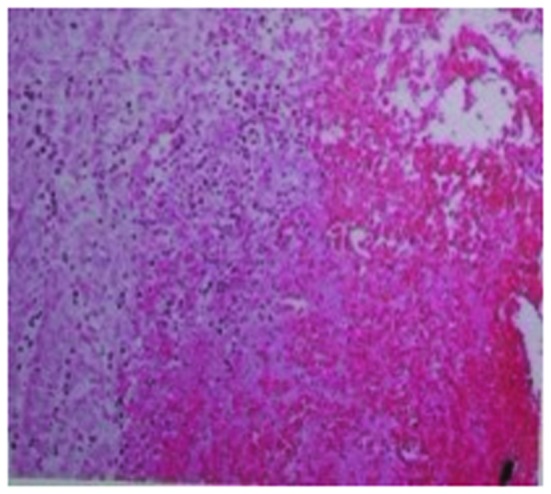
Case 1: A thrombus was identified in the lumen of the blood vessel.

The blood, urine, stool routine, liver and kidney function, electrocardiogram, chest radiography, arteriovenous color Doppler ultrasound of bilateral extremities and foot X-ray results were all normal. The hepatitis B test results were as follows: HBsAg^+^, HBeAb^+^ and HBcAb^+^. The immunity test revealed complement component 3 (C3) levels of 0.49 g/l. The T lymphocyte subsets and NK cell counts included CD3^+^, 85%; CD4^+^, 49%; CD8^+^, 27%; and NK cells, 8%. The T lymphocyte subset H/S ratio was 1.81. The rheumatism, antinuclear antibody spectrum and antineutrophil cytoplasmic antibody test results were negative, as were the bacterial and fungal cultures. Following pathological examination of the left shank, collagen-based dermis was identified, without evident proliferation of the epidermis. An infiltration of inflammatory cells was observed in the appendage, the vein wall and fat tissues. A large blood vessel was identified in the injured tissue. Moreover, inflammation was evident in the inner membrane and the wall of the blood vessel and a thrombus was identified in the lumen of the blood vessel (Fig. 4).

#### Case 2

The blood, urine, routine stool, liver and kidney function, electrocardiogram, chest radiography, arteriovenous color Doppler ultrasound of bilateral extremities and foot X-ray results were all normal. The rheumatism, antinuclear antibody spectrum and antineutrophil cytoplasmic antibody test results were all negative. The T lymphocyte subsets and NK cell counts were as follows: CD3^+^, 67%; CD4^+^, 38%; CD8^+^, 26%; and NK cells, 12%. Additionally, the T lymphocyte subset H/S ratio was 1.46, and the bacterial and fungal culture tests were negative.

#### Diagnosis

Two patients were 29 and 33 year-old male smokers, with lower limb involvement. In case 1, a 3×3 cm ulcer was identified on the skin of the patient’s right foot, with partial tendon exposure and yellow purulent discharge. Furthermore, the right fifth toe was swollen and infected. The skin temperature of the right foot was low, the sensitivity to touch was reduced and the pulse at the top of the right foot was weak. In case 2, pathological examination of the left shank revealed collagen-based dermis, as well as an infiltration of inflammatory cells in affiliated tissue, vein wall and fat tissues. A large blood vessel, with a thrombus in the lumen, was observed in the injured tissue. According to the above features, the two patients were diagnosed with TAO.

#### Treatment

**Figure 5.**
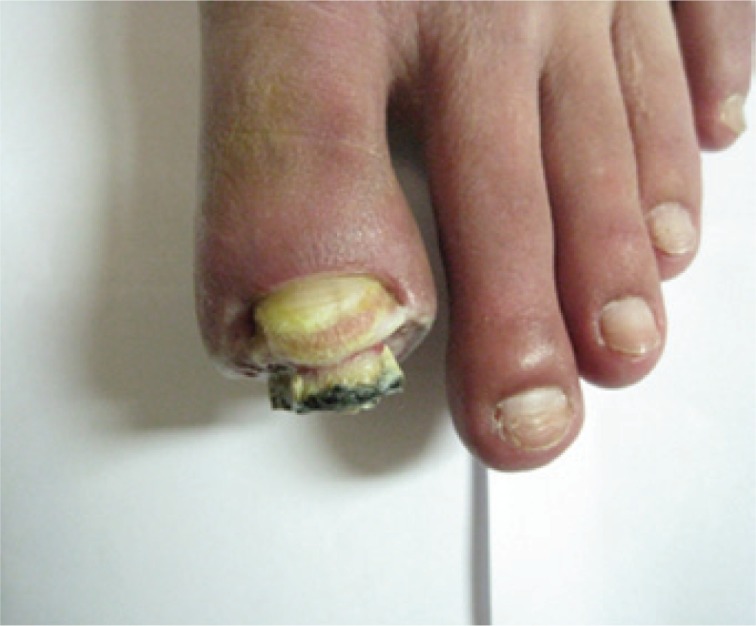
Case 2: The ulcerated area on the first toe of the left foot has reduced in size, and a reduction in secretions and inflammation is evident.

**Figure 6.**
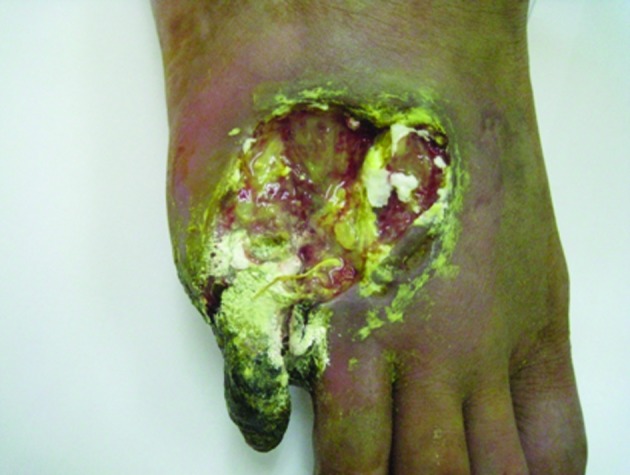
Case 1: The Ulcer on top of right foot has increased in size to 6.5×8 cm. The exposed tendon is broken and the level of yellow purulent discharge has decreased.

Treatments for TAO included stopping smoking, resting and keeping warm. To promote blood circulation, the patient was treated with compounds such as Danshen Dripping Pills, Fufang Danshen Diwan and Salvia tetramethylpyrazine. The patients were also treated with fibroblast growth factor to promote epidermal growth and Bayaspirin enteric-coated tablets to reduce platelet aggregation. The polysaccharide nucleic acid fraction of Bacillus Calmette-Guérin and compound glycyrrhizin tablets were taken to improve immune function. Following treatment, the patient in case 2 had reduced pain levels in the left foot. The ulcer on the first toe of the left foot had decreased in size, with a reduction in pus secretions and inflammation (Fig. 5). The patient in case 1 demonstrated a reduction in pus secretion from the ulcer; however, the area of the ulcer had increased, spreading to the fifth toe with gangrene. A tendon had become exposed on the right foot, which was broken and inducing severe pain (Fig. 6).

## Discussion

The occurrence of TAO has been increasing and has a marked effect on the health and quality of life of affected individuals (11). The disease has a high morbidity in China and frequently occurs in smokers <45 years of age (11). The cause of TAO remains unknown; however, use or exposure to tobacco is related to the onset and progression of the disease. Nicotine and carboxyhemoglobin may cause damage to the structure of endothelial cells and inflammation of the vascular endomembrane, resulting in thrombus formation. Thus, an individual’s smoking history is an important basis for the diagnosis of TAO (6,12). The patients in cases 1 and 2, who were brothers, had a long-term history of smoking. Furthermore, it was identified that the levels of adhesion molecules in the plasma of the two patients, such as intracellular adhesion molecule 1 (ICAM-1), vascular cell adhesion molecule 1 (VCAM-1) and inflammatory factor, were significantly higher than that in healthy individuals. Therefore, it was concluded that an inflammatory response is significant in the progression of TAO (13,14). Studies have demonstrated that anti-collagen, -elastic protein, -endothelial cell, -cardiolipin and -neutrophil cytoplasmic antibody levels increase during the active period of TAO (15,16). Therefore, it has been suggested that hypersensitivity responses and autoimmune disorders are involved in the progression of TAO. However, the two patients in the current case report had normal autoimmune antibodies.

Erythema nodosum is a type of skin inflammation with the histopathology of small vessel vasculitis in the deep dermis and panniculitis. It is characterized by painful nodules that are located on the lower extremities. Erythema nodosum may resolve spontaneously within 3–5 weeks (17). Takanashi *et al* previously studied one patient with TAO who had erythema nodosum and a black reticulum plaque in the early stages of the disease. An early biopsy indicated that numerous mono-nuclear cells had subcutaneously infiltrated the dermis and perivascular areas. Following treatment with prednisolone, the erythema nodosum and livedo reticularis improved; however, the necrosis and ulceration of the lower limbs repeatedly occurred. The patient underwent amputation due to a severe ulcer 1.5 years later. The postoperative pathological examination demonstrated anterior tibial artery inflammation and the formation of a thrombus in accordance with TAO. Therefore, symptoms of erythema nodosum and livedo reticularis may suggest a diagnosis of TAO (18).

The present case report identified two brothers who had painful nodules and erythema in the leg. Administration of immunosuppressive agents resulted in a reduction in the symptoms of TAO, before the symptoms repeatedly occurred. The pathological biopsy demonstrated that the vessels had undergone inflammatory changes. At a later stage, the erythema was significantly reduced, although increased ulceration was present. Both patients demonstrated reduced touch sensitivity. A primary biopsy identified a thrombus in the vascular cavity; therefore, the diagnosis of TAO was confirmed. TAO is rarely identified in individuals of the same family. However, due to the present two cases, we propose that familial factors ought to be considered when conducting TAO treatment. Wysokinski *et al* described two brothers who were simultaneously diagnosed with TAO. It was considered that there was a correlation between a gene mechanism and TAO (19). Therefore, a complete family history may be required in clinical practice. Chen *et al* performed a gene polymorphism analysis in 131 patients with TAO and 227 healthy individuals, in order to test the promoters of HLA-DPB1, -DRB1 and -B, and CD14. The genotype frequency of CD14 TT was significantly higher in TAO patients than in the healthy controls. Chen *et al* suggested that at least three genes were associated with the occurrence of TAO, and that TAO may be regulated by genes, with the involvement of innate and acquired immunity(20).

It has been demonstrated that the most effective way to prevent and treat TAO is to stop smoking (2). Amputation and vascular reconstruction are not routine treatments, due to chronic ischemia of the distal extremities. Olin *et al* revealed that 94% of patients did not undergo amputation subsequent to stopping smoking, whereas 43% of patients who smoked underwent amputation surgery (6). Adjuvant drugs, including iloprost, prostaglandin vasodilator agents, antiplatelet drugs, aspirin and vasodilator agents (such as, α-adrenergic blocking agents and calcium antagonists), may effectively reduce the symptoms of pain associated with TAO. Vascular endothelial growth factor aids the recovery of ischemic ulcers. In addition, spinal cord stimulation and surgical sympathectomy have also been reported to be beneficial; however; the long-term effects are yet to be confirmed (2,21).

The two patients presented in this case report stopped smoking due to the diagnosis of TAO. Following treatment with vasodilator agents, microcirculation improvement interventions and surgical excision, the ulcer on the left foot of the patient in case 2 decreased. The patient in case 1 developed dry gangrene and partial ischemia in the right foot. This patient underwent amputation of the fifth toe and skin grafting treatment.
